# Police Bias and Low Relatability and Diet Quality: Examining the Importance of Psychosocial Factors in Predominantly Black Communities

**DOI:** 10.1007/s11524-023-00785-0

**Published:** 2023-10-04

**Authors:** Andrea S. Richardson, Rebecca L. Collins, Rachel M. Burns, Jonathan Cantor, Sameer M. Siddiqi, Tamara Dubowitz

**Affiliations:** 1 Department of Behavioral and Policy Sciences, RAND Corporation, Pittsburgh, PA 15213 USA; 2 Department of Behavioral and Policy Sciences, RAND Corporation, Santa Monica, CA 90401 USA; 3 Department of Economics, Statistics, and Sociology, Pittsburgh, PA 15213 USA; 4 Department of Economics, Statistics, and Sociology, RAND Corporation, Santa Monica, CA 90401 USA; 5 Department of Behavioral and Policy Sciences, RAND Corporation, Arlington, VA 22202 USA

**Keywords:** Dietary quality, Black Americans, Police, Disparities, Stress, Social status, Community connectedness

## Abstract

How police bias and low relatability may contribute to poor dietary quality is poorly understood. In this cross-sectional study, we analyzed data from 2021 from a cohort of *n* = 724 adults living in predominantly Black communities in Pittsburgh, Pennsylvania; these adults were mostly Black (90.6%), low-income (median household income $17,500), and women (79.3%). We estimated direct and indirect paths between police mistrust and dietary quality (measured by Healthy Eating Index (HEI)-2015) through perceived stress, community connectedness, and subjective social status. Dietary quality was poor (mean HEI-2015 score was 50) and mistrust of police was high: 78% of participants either agreed or strongly agreed that something they say might be interpreted as criminal by the police due to their race/ethnicity. Police bias and low relatability was associated with lower perceived social status $$\beta$$=  − 0.03 (95% confidence interval [CI]: − 0.05, − 0.01). Police bias and low relatability was marginally associated with low dietary quality β =  − 0.14 (95% CI: − 0.29, 0.02). Nineteen percent of the total association between police bias and low relatability and lower dietary quality β =  − 0.16 (− 0.01, − 0.31) was explained by an indirect association through lower community connectedness, or how close respondents felt with their community $$.$$ Police bias and low relatability may play a role in community connection, social status, and ultimately dietary disparities for Black Americans. Addressing police bias and low relatability is a continuing and pressing public health issue.

## Introduction

Black Americans bear higher rates of nutrition-related disease than white Americans [1] and are also more likely to reside in socioeconomically disadvantaged neighborhoods with high crime rates [2]. While much of the built and social conditions of urban neighborhoods across the USA are the result of historical processes rooted in racism [3], indicators including education, employment, housing quality, and poverty rates have helped to characterize and define socioeconomically disadvantaged neighborhoods [4]. Neighborhood disinvestment has reinforced structural health barriers, including crime [5], poverty [6], and limited food and physical activity resources [5, 7–10].

Living in neighborhoods with increased crime and lower socioeconomic conditions can put Black Americans at risk of interacting with police [11]. Unfair treatment of Black Americans by police is a form of institutional racism that has been associated with poor health [12]. Direct and collateral harms of police brutality are both physical and psychological, and consequences exacerbate social and economic hierarchies. Consequently, Black Americans face situations of disempowerment with limited recourse when treated unjustly. Several responses have been hypothesized in the literature [13, 14], Aside from anger, Black individuals’ mental health may be strained, and they may feel stress, diminished trust in institutions, and loss of community connection and social status. As Sewell et al. proposed, this can happen “through the production of social emotions that compromise feelings of belongingness and safety and enhance distrust of and resentment toward police” [14]. Devylder et al. proposed a framework that distinguishes police violence from other forms of violence and trauma because of the unique characteristics of police (e.g., state sanctioned), their interactions with civilians, and consequent assaults to mental health [15]. In this work, there are separate pathways outlined where four factors—weapon access, state-sanctioned violence, perceived racial and class biases, and incarceration risk—likely increase mental health strain through immediate impacts of violent incidents. Three other factors—pervasive presence, limited recourse, and stigma of reporting police violence—are related to chronic and long-lasting exposure to police violence that undermine mental health by impeding coping and recovery. An overarching feature of police is that they are armed within the context of persistent historical racism [16] that exacerbates power dynamics and increases the likelihood for aggressive police interaction. Very little work to our knowledge, however, has made the connection between these factors and dietary outcomes.

Police violence and health are related across the life course. Youth is a critical period of development and increasing research demonstrates how direct and indirect exposure to police interactions in detrimental to youth of color [11]. Studies using data from the national Fragile Families and Child Wellbeing Study have reported associations between direct and indirect police contact and worse reported health, emotional distress and posttraumatic stress symptoms for Black and Hispanic youth [17–19]. Emerging Black women may be particularly vulnerable to impacts of police force on depression and anxiety [20]. A systematic review reported an association between police exposure and adverse mental health, sexual risk behaviors, and substance use for Black youth. These findings support the detrimental impacts of police brutality as communities of color, beginning in youth.

Consequences of police brutality likely accumulate over time as people age. Interactions with police have been associated with poor health in Black adults, including diabetes, high blood pressure, and obesity [14]. Given nutrition-related diseases linkages with police brutality, dietary quality may be one mechanism through which police brutality impacts health. Dietary behaviors are driven by psychosocial forces beyond physiological energy balance [21]. For example, chronic stress exposure may increase consumption of “comfort food” as a means to self-medicate [22]. Stress is the human response to the perception of and response to threat resulting in unwanted distress [23] and contributes to excess intake of alcohol, high fat, and sugary foods and beverages [24].

Stress associated with aggressive policing and surveillance may strain psychosocial resources, such as community connection and perceived status, that support healthy dietary behaviors [25]. Social connection is when people have stable supportive relationships with their community and is a determinant of health [26] that operates through perceptions and processing of social experiences influencing health behaviors [27]. Despite longitudinal evidence linking social connection with multiple health outcomes including cardiovascular disease, it is understudied in health research. Social connection may be undermined by stress, police bias, and low relatability.

Exposure to police brutality and constant surveillance can also harm peoples’ perception of their social standing where acceptance of negative cultural stereotypes can lead to unfavorable self-evaluations [28]. Lower perceived social status has been associated with dietary behaviors, such as dysregulated eating [29] and excess energy intake [30]. In sum, aggressive and biased policing and decreased relatability can increase stress and harm stress-related psychosocial resources, such as community connection and perceived status, that support healthy dietary behaviors. No studies to our knowledge have quantified whether and how police bias and low relatability might be associated with dietary outcomes.

We hypothesized that police bias and low relatability influences Black Americans’ dietary quality. Higher levels of bias and reduced relatability are associated with lower perceived social status, lower sense of community connectedness, and higher stress. Each of these may in turn tax constrained resources available to engage in healthy dietary habits. We examined associations between police bias and low relatability and dietary quality, with social status, community connectedness, and stress as potential explanatory pathways, in a cohort of predominantly Black American older women living in low-income Pittsburgh, Pennsylvania (PA) neighborhoods.

## Methods

### Participants

We analyzed data from the most recent wave of The Pittsburgh Hill/Homewood Research on Shopping and Health (PHRESH) study data (collected May–November 2021). PHRESH is a quasi-experimental longitudinal study with the original intent of estimating the effects of opening a full-service supermarket on diet and BMI in a low-income, predominantly Black neighborhood in Pittsburgh, Pennsylvania [31]. A random sample of households from two sociodemographically similar neighborhoods (the Hill District and Homewood) was enrolled in 2011 and has been followed since. Trained interviewers enrolled study participants via door-to-door recruitment in 2011, and since have administered waves (2014, 2016, 2018, and 2021) of surveys that have captured neighborhood perceptions, psychological measures of social status, community connectedness, stress, perception of relatability to and bias of police, and dietary intake. The adult ($\ge 18$ years old) primary food purchaser in each household was asked to enroll resulting in a sample of predominantly older women because they were the primary food shopper home during recruitment. Sampling approach, recruitment, and eligibility have previously been described [31]. A total of 724 participants completed the PHRESH 2021 survey which we used for all measures in this study. All study protocols were approved by the institution’s Institutional Review Board.

### Dependent Variable

At the interview, dietary intake data for (24-h recalls) were interviewer-assisted collected and analyzed using Automated Self-Administered 24-h (ASA24) Dietary Assessment Tool, version (2020), developed by the National Cancer Institute, Bethesda, MD, USA. Participants were asked to report all food and drinks they consumed in the previous 24 h. A second dietary recall was administered by phone within 7–10 days of the initial interview and responses averaged across the two. We used the simple HEI scoring algorithm [32] to derive HEI-2015 scores as a sum of 13 component scores [33], each of which measures compliance with a different aspect of the Dietary Guidelines. Each component is assigned a standard for achieving a maximum score. The components are summed to a single per person score for each wave based on the average of the two recalls. Scores range from 0 to 100; higher score indicates better dietary quality.

### Independent Variable

Participants responded to 8 survey questions about police bias and relatability. The responses for each of the measures were based on a Likert scale that ranged from 1 (strongly disagree) to 5 (strongly agree).

- You feel safe around the police (reverse-coded)
- You personally trust the police (reverse-coded)
- Police officers will treat you differently because of your race/ethnicity
- Police officers will judge you based on your race/ethnicity
- The police act based on personal prejudices or biases
- Something you do might be misinterpreted as criminal by the police due to your race/ethnicity
- Something you say might be misinterpreted as criminal by the police due to your race/ethnicity
- The police suspect you of being a criminal because of your race/ethnicity

Questions were adapted from a six-city pilot effort to restore relationships between police and members residing in high-crime communities with strained police-community relations [34]. The pilot survey included questions to capture legitimacy, procedural justice, racial bias, relatability to police, and applied principles of community policing. Here, we focused on police bias and relatability measures which reflected residents’ negative perceptions in the pilot. We constructed a measure of police bias and low relatability by summing across responded values, after reverse coding questions 1 and 2. The Chronbach alpha is a measure of internal consistency, which was 0.89, demonstrating the items are closely related. The police bias and relatability score have a possible range of 1–40 and higher value represents greater police bias and low relatability.

### Pathways

#### Perceived Stress

We used the validated Perceived Stress Scale-4 [35]. Residents were asked four questions with a past month reference period: “(1) how often have you felt that you were unable to control the important things in your life?; (2) how often have you felt confident about your ability to handle your personal problems?; (3) how often have you felt that things were going your way?; and (4) how often have you felt difficulties were piling up so high that you could not overcome them?” Response categories of never, almost never, sometimes, fairly often, and very often were assigned values of 0 through 4. Questions 2 and 3 were reverse coded and values across the four questions were summed so that higher values indicate more stress.

#### Community Connectedness

**Fig. 1:**
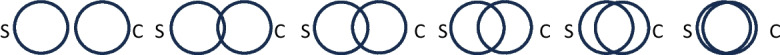
Community connectedness

We used a single item (Fig. 1) that we adapted from a measure developed to measure how close the respondent feels with their community or “inclusion of other in self” (‘community connectedness’ hereafter) [36]. Respondents were shown six pairs of circles where one circle represents the self and the other the community. They were asked to select the pair that best describes their relationship with their larger community (S = self; C = larger community). The response options were scored from 1 to 6 with the higher score reflecting the most community connectedness.

#### Perceived Social Status

We used a single-item Macarthur subjective social status measure that assesses a person’s perceived rank relative to others in their group [37]. Participants responded to a figure of a ladder with 10 rungs, and interviewers explained that the ladder represented where people stand in society: “At the bottom are the people who have the least money, least education, and least respected jobs or no job. The higher up you are on this ladder, the closer you are to the people at the very top, and the lower you are, the closer you are to the people at the very bottom. Where would you place yourself on this ladder?” The higher the value on the scale the higher the social status. The rungs were assigned values from 1 to 10 where higher values reflect higher reported social status.

### Covariates

Covariate data was collected with trained data collector-administered surveys of respondents. We adjusted for sociodemographic characteristics associated with police bias and low relatability, proposed pathways and/or dietary outcomes [38]: age, gender, household income, educational attainment greater than high school versus less than, any children in the household, married/living with a partner versus not.

To capture neighborhood characteristics that could be associated with police perceptions and dietary behaviors we adjusted for neighborhood (Hill District, Homewood, or other), satisfaction which was assessed with the question, “All things considered, would you say you are very satisfied, satisfied, dissatisfied, very dissatisfied, or neutral—neither satisfied or dissatisfied with your neighborhood as a place to live?” We also adjusted for perceived neighborhood safety which was assessed using four items (e.g., ‘You feel safe walking in your neighborhood during the day,’ ‘You feel safe walking in your neighborhood during the evening,’ ‘Your neighborhood is safe from crime’, ‘Violence is a problem in your neighborhood’). Response options for each item ranged from 0 (strongly disagree) to 4 (strongly agree). Items were reverse coded as necessary. Higher scores indicate higher perceived safety.

### Statistical Analysis

We calculated means, standard deviations (continuous variables) and percentages (categorical variables). We estimated Pearson pairwise correlations of police bias and low relatability, dietary quality (Healthy Eating Index or HEI-2015), and proposed pathways (stress, community connectedness, and social status). We estimated descriptive statistics with Stata 17.0 (StataCorp, College Station, TX). We sought to understand to what extent community connectedness, stress, and social status explain association between *police bias and low relatability* and dietary behaviors. In this cross-sectional study, we examined direct and indirect pathways from police bias and low relatability to HEI-2015 through hypothesized paths, using structural equation modeling (SEM). SEM is a pathway-based approach that can handle multi-equation models, and allows estimation among multiple effects transmitted over combinations of paths simultaneously [39]. We used Mplus version 7.11 [40] with robust maximum likelihood estimation that allows for missing data. We allowed for a direct pathway from *police bias and low relatability* to dietary quality. A root mean square error of approximation (RMSEA) < 0.06 [41] and comparative fit index (CFI) values > 0.95 [41] imply the model fits the data well.

### Sensitivity Analyses

Given the police racial targeting of Black people, we excluded participants who reported that they were neither Black nor mixed-Black.

## Results

Among the total sample (*N* = 724), 548 (76%) were Black and mixed-Black women who were on average 62 years old (Table 1). More than half (53%) had attained greater than a high school diploma, and median annual household income was $17,500. Most did not live with children or a partner. Two thirds were satisfied with their neighborhood although perceived safety was about 3 out of a possible score of 5. To provide context, 59% agreed or strongly agreed that violence is a problem in their neighborhood while 61% also agreed or strongly agreed that they felt safe walking in their neighborhood.

**Table 1:** Participant characteristics (*N* = 724) in 2021, Pittsburgh, Pennsylvania

Demographic characteristics	
Age in years, mean (SD)	61.5 (14.1)
Race/ethnicity, no. (%)	
American Indian or Alaska Native	3 (0.4)
Asian	1 (0.1)
Black	656 (90.6)
Black mixed	29 (4.0)
White	9 (1.2)
Other^1^	22 (3.0)
Missing	4 (0.6)
Gender, no. (%)	
Female	574 (79.3)
Male	149 (20.6)
Non-binary	1 (0.1)
Highest education attained, no. (%)	
Greater than high school	382 (52.8)
Annual household income, median (25^th^, 75^th^ percentiles)	17,500 (7,500, 35,000)
Any children in household	140 (19.3)
Married/living with partner versus not, no. (%)	106 (14.6)
Missing	4 (0.6)
Neighborhood, no. (%)	
Hill district	428 (59.1)
Homewood	217 (30.0)
Other	79 (10.9)
Neighborhood satisfaction- No. (%)	488 (67.4)
Perceived neighborhood safety, mean (SD)	3.1 (0.8)
Outcome	
Healthy Eating Index-2015, mean (SD)	50.0 (12.2)
Exposure	
Police bias and lack of relatability score (range 0–40), mean (SD)	27.9 (6.0)
You feel safe around the police	
Strongly disagree	34 (4.7)
Disagree	137 (18.9)
Neither agree nor disagree	116 (16.0)
Agree	390 (53.9)
Strongly agree	42 (5.8)
Missing	5 (0.4)
You personally trust the police	
Strongly disagree	37 (5.1)
Disagree	229 (31.6)
Neither agree nor disagree	174 (24.0)
Agree	258 (35.6)
Strongly agree	20 (2.8)
Missing	6 (0.8)
Police officers will treat you differently because of your race/ethnicity	
Strongly disagree	11 (1.5)
Disagree	104 (14.4)
Neither agree nor disagree	84 (11.6)
Agree	357 (49.3)
Strongly agree	159 (22.0)
Missing	9 (1.2)
Police officers will judge you based on your race/ethnicity	
Strongly disagree	12 (1.7)
Disagree	91 (12.6)
Neither agree nor disagree	83 (11.5)
Agree	386 (53.3)
Strongly agree	146 (20.2)
Missing	6 (0.8)
The police act based on personal prejudices or biases	
Strongly disagree	5 (0.6)
Disagree	96 (13.3)
Neither agree nor disagree	114 (15.8)
Agree	383 (52.9)
Strongly agree	109 (15.1)
Missing	17 (2.4)
Something you do might be misinterpreted as criminal by the police due your race/ethnicity	
Strongly disagree	9 (1.2)
Disagree	85 (11.7)
Neither agree nor disagree	55 (7.6)
Agree	431 (59.5)
Strongly agree	137 (18.9)
Missing	7 (1.0)
Something you say might be misinterpreted as criminal by the police due your race/ethnicity	
Strongly disagree	8 (1.1)
Disagree	80 (11.1)
Neither agree nor disagree	58 (8.0)
Agree	441 (60.9)
Strongly agree	126 (17.4)
Missing	11 (1.5)
The police suspect you of being a criminal because of your race/ethnicity	
Strongly disagree	11 (1.5)
Disagree	140 (19.3)
Neither agree nor disagree	99 (13.7)
Agree	368 (50.8)
Strongly agree	100 (13.8)
Missing	6 (0.8)
Mediators	
Community connectedness—median (25^th^, 75^th^ percentiles)	3 (1, 4)
Subjective social status, mean (SD)	5.2 (1,7)
Perceived stress—median (25^th^, 75^th^ percentiles)	4 (2, 6)

^1^Caribean, Creole, Dominican, Hebrew Israelite, Indian, Jamaican, Somalia Bantu Spanish, mixed race 724 (100.0)

**Fig. 2:**
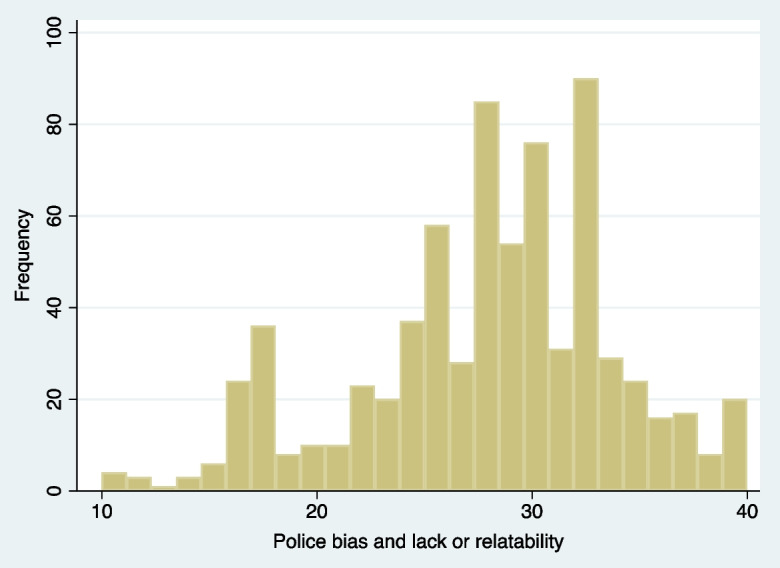
Frequency histogram of police bias and low relatability score in 2021, Pittsburgh, Pennsylvania

Participants’ average HEI-2015 score was 50 out of a possible score of 100. Participants’ police bias and low relatability score was normally distributed (Fig. 2), with an average score of 27 out of a possible 40 score. Over half (61%) of participants strongly disagreed or disagreed or were ambivalent that they personally trusted the police. Between 65 and 78% of participants strongly agreed or agreed that police would treat, judge, misinterpret them as criminal because of their race, and act on prejudice and bias. However, almost 60% agreed or strongly agreed that they felt safe around the police. This positive police perception was larger than observed in the six-city study where 38% reported feeling safe around the police [34]. Participants reported feeling an average sense of community connectedness with a median score of 3 out of a possible 6. Similarly, participants felt their status was about the middle of the 10-runged ladder which was slightly lower than what Black women in the Jackson Heart Study reported (mean = 6.4) [42]. However, participants reported feeling relatively lower stress than a sample of middle-aged women (mean = 5.2) [43].

In pairwise correlations, community connectedness was positively correlated with dietary quality (HEI-2015 score), and police bias and low relatability was negatively correlated with belonging and social status (Table 2).

**Table 2:** Pearson correlations of participants’ community connectedness, social status, stress, and police bias and lack of relatability score in 2021, Pittsburgh, Pennsylvania

Rho/*P*-value	Healthy Eating Index-2015	Community connectedness	Subjective social status	Perceived stress	Police bias and lack of relatability score
HEI-2015 total scores	1				
Community connectedness	0.16	1			
	0.00				
Subjective social status	0.06	0.18	1		
	0.09	0.00			
Perceived stress	− 0.10	− 0.12	− 0.19	1	
	0.01	0.00	0.00		
Police bias and lack of relatability score	− 0.06	− 0.10	− 0.08	0.01	1
	0.12	0.01	0.03	0.77	

**Fig. 3:**
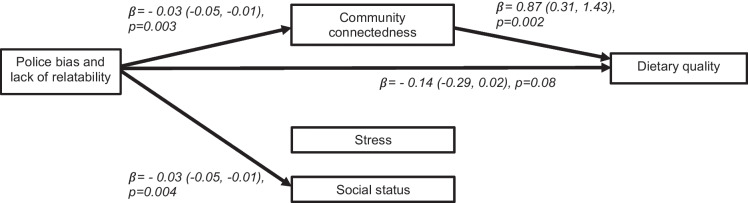
Structural equation model of direct and indirect paths from police bias and low relatability to dietary quality in 2021 through community connectedness, stress, and social status, Pittsburgh, Pennsylvania

The SEM model fit the data well (RMSEA = 0.02, CFI = 0.96). Police bias and low relatability was marginally associated (*p* < 0.10) with low dietary quality $\beta$=  − 0.14 (95% CI: − 0.29, 0.02) (Fig. 3) which suggests that an increase in one point on the police bias and low relatability score reduces the HEI-2015 by 0.14 points. An indirect path through lower community connectedness explained 21% of the direct association between police bias and low relatability with lower dietary quality $\beta$=  − 0.03 (95% CI: − 0.05, − 0.01). Healthy diet was positively associated with community connectedness $\beta$= 0.87 (95% CI: − 0.31, 1.43). Indirect paths through stress and social status did not explain the association between police bias and low relatability and diet. While police bias and low relatability was associated with lower social status $\beta$=  − 0.03 (95% CI: − 0.05, − 0.01), this did not explain the police bias and low relatability and diet correlation. Stress was neither associated with police bias and low relatability nor dietary quality. All model estimates are presented in Table 3.

**Table 3:** Estimates from structural equation models examining associations between independent variables and HEI-2015 score

Dependent/independent variables	*β* (95% confidence interval)
Sense of belonging	0.87 (0.31, 1.43)
Subjective social status	− 0.06 (− 0.59, 0.47)
Police bias and lack of relatability	− 0.14 (− 0.29, 0.02)
Perceived stress	− 0.19 (− 0.49, 0.12)
Neighborhood safety	− 0.51 (− 1.68, 0.67)
Neighborhood satisfaction	− 0.46 (− 2.48, 1.56)
Gender: non-binary	− 11.76 (− 34.69, 11.17)
Gender: male	1.79 (− 0.38, 3.95)
Educational attainment greater than high school	2.86 (0.98, 4.74)
Neighborhood: Homewood	2.37 (0.40, 4.35)
Neighborhood: other	0.32 (− 2.55, 3.19)
Age (years)	0.04 (− 0.03, 0.12)
Married/living with partner	− 0.76 (− 3.36, 1.84)
Any children in household	− 1.38 (− 3.91, 1.15)
Annual household income	0.77 (0.27, 1.26)
Sense of belonging	
Police bias and lack of relatability	− 0.03 (− 0.05, − 0.01)
Annual household income	0.10 (0.04, 0.16)
Subjective social status	
Police bias and lack of relatability	− 0.03 (− 0.05, − 0.01)
Annual household income	0.20 (0.14, 0.25)
Perceived stress	
Police bias and lack of relatability	0.01 (− 0.02, 0.05)
Annual household income	− 0.23 (− 0.33, − 0.12)

### Sensitivity Analyses

When restricting to the Black and mixed-Black participants the results were very similar although slightly attenuated. An indirect path through lower community connectedness explained 13% of the direct association between police bias and low relatability with lower dietary quality $\beta$=  − 0.02 (95% CI: − 0.05, 0.00).

## Discussion

Health effects of aggressive policing on Black Americans are understudied. Police violence is not well tracked [44] and underlying processes through which exposure to police brutality manifests into health disparities are poorly understood. Residents of high crime neighborhoods navigate economic hardship and dangers of crime while also using strategies to avoid threats posed by police who are supposed to protect them but can also sanction them [45]. Balancing stresses carries costs and “coping fatigue” [46]. We found evidence that police bias and low relatability may undermine dietary quality through reduced community connectedness. Furthermore, police bias and low relatability may be detrimental to Black American’s perceived social status. To our knowledge, our study is the first to associate police bias and low relatability with dietary behaviors through mediating processes.

PHRESH participants live in Pittsburgh neighborhoods with high crime, so likely have frequent interactions with police. Pittsburgh spans 58.3 mile^2^ and in 2021, 1342 violent crimes occurred, which translates to about 23 violent crimes per mile^2^ [47]. The 2021 violent crime rate was 55 crimes per mile^2^ in the Hill District (76 crimes within 1.37 mile^2^) and 112 violent crimes per mile^2^ in Homewood (163 crimes within 1.45 mile^2^). Communities with high crime are also burdened with structural barriers to health, such as lack of high-quality housing, limited food and physical activity resources, and poor economic and educational resources. Racist policies and practices created and continue to reinforce the structural factors that harm Black communities and limit their access to socioeconomic mobility and health. Our participants reported greater prevalence of police bias and low relatability than in the six-city study [34] where between 47 to 56% agreed or strongly agreed police were biased compared to 68% to 74% in our study. Perceived police bias could arise in response to the structural racism Black communities face daily and in recognition that policing is an institution created by white people. Police bias and low relatability may be related to aggressive policing in high crime neighborhoods, but they may also be related to structural racism that is at the root of the neighborhood poverty and disinvestment that undermine healthy behaviors.

While crime data are reported by the Pittsburgh City Police Department, specific police violence is not yet reported nationally. However, the Federal Bureau of Investigation recently began collecting use-of-force data from reporting law enforcement agencies. These occurrences were previously not reported by law enforcement and this new effort aims to increase transparency. Within Pittsburgh, there have been 65 police-involved fatalities of Black residents since 2000 compared to 13 fatalities of white residents [48]. In contrast, most of Pittsburgh’s population is white (65%) and only a minority Black (23%). Our study sample reported an average HEI-2015 score of 50 which is relatively lower than the national average in 2017–2018 of 52.7 but similar to the average among non-Hispanic Black (50.8) [49].

Our sample is largely women. While women may not interact with police as much as men, their lives are often impacted by policing and imprisonment of their family and loved ones [50]. Women are usually the first person contacted by an arrested person [50]. In addition, losing a household member to incarceration can increase women’s caretaking responsibilities, financially and otherwise [51]. Women have reported frustration and fear of police neglect and misbehavior, and having an incarcerated family member has been associated with women’s reduced cardiovascular health [52].

Our findings identified an indirect association between police bias and low relatability and dietary quality. Dietary behaviors are potent and modifiable causes of nutrition-related disease [53]. Personal dietary choices and preferences are complex and can be influenced by social forces. Our findings suggest one pathway may exist between police bias and low relatability and reduced community connectedness. These results support other research that identified associations with police brutality and nutrition-related diseases such as obesity, hypertension, and obesity [14].

We also found that greater police bias and low relatability was associated with participants’ low rated social status. This aligns with research where Black Americans internalize racism by subscribing to a sense of innate inferiority [54]. While internalized racism has been associated with alcohol consumption [55], we did not find evidence that it was associated with dietary behaviors in our participants. Future studies should examine if there is a relationship between police bias and low relatability and social status within other populations and localities.

We did not identify an association between police bias and low relatability and stress whereas other studies have found associations between police brutality and adverse mental health outcomes [56]. In a quasi-experimental, population-based study of 103,710 Black Americans, each additional police killing of an unarmed black American in 2013–2015 was associated with 14 additional poor mental health days among black but not white American respondents [56]. The largest effects on mental health occurred in the 1–2 months after exposure, not before police killings. However, the lack of association between police bias and low relatability score and stress in our study suggests that the metric is not biased by respondents’ anxious traits. However, associations between police bias and low relatability, belonging and social status increase the possibilities of more complex causal relationships than can be identified through this cross-sectional design. Quantitative study designs that allow causal inference designs that explore these associations more directly are needed. Furthermore, future studies using qualitative or mixed methods approaches should explore this topic in more detail to improve our understanding of how police violence may influence health behavior.

Our study has limitations, and findings should be interpreted with caution. The study is cross-sectional. However, our participants’ perceptions of police bias and relatability, stress, subjective social status, and community connectedness have likely developed over time. Future research that leverages temporal changes in police and resident relationships, responses, and health behaviors should attempt to replicate our findings. Our survey did not capture personal experiences with police interactions nor any collateral pain participants may have suffered from loved ones’ encounters with law enforcement. However, we were able to estimate direct and indirect paths using rigorous dietary data and detailed perceptions of police bias and relatability, among an understudied population. Untested indirect pathways may explain more of the association between police bias and low relatability and diet than we were able to identify. For example, biased policing and resulting in eroded trust of police may undermine trust in federal dietary guidelines issues by medical and public health institutions. The estimated effect size of the direct and indirect associations was small in relation to the HEI-2015 score. Our sample included few men who likely have very different perceptions of police given that they are they are more likely to be targeted and victimized by the police than women [45]. Lacking men’s contribution to this study could have biased our findings either away from or toward the null. Finally, our results are not generalizable to other locations or populations. Cities across the country differ significantly with respect to histories of populations of color and police culture. During the Great Migration, many Black people left southern areas while foreign immigrants were finding residence in large northern cities. Interactions from shifting demographics may underlie place-based cultures that influence policing approaches and perceptions.

Black American’s are disproportionately burdened by nutrition-related disease and racially discriminatory policing. Police bias and low relatability from Black peoples’ unfair treatment may be one contributor to health burdens among Black people, including poor dietary quality.
